# Release of Mannoproteins during *Saccharomyces cerevisiae* Autolysis Induced by Pulsed Electric Field

**DOI:** 10.3389/fmicb.2016.01435

**Published:** 2016-09-12

**Authors:** Juan M. Martínez, Guillermo Cebrián, Ignacio Álvarez, Javier Raso

**Affiliations:** Tecnología de los Alimentos, Facultad de Veterinaria, Instituto Agroalimentario de Aragón, Universidad de ZaragozaZaragoza, Spain

**Keywords:** pulsed electric fields, mannoproteins, autholysis, *Saccharomyces cerevisiae*, winemaking

## Abstract

The potential of the application of pulsed electric fields (PEF) to induce accelerate autolysis of a commercial strain of *Saccharomyces cerevisiae* for winemaking use was evaluated. The influence of PEF treatments of different intensity (5–25 kV/cm for 30–240 μs) on cell viability, cytoplasmic membrane permeabilization and release of mannoproteins and compounds absorbing at 260 and 280 nm has been investigated. After 8 days of incubation at 25°C the Abs_600_ of the suspension containing the control cells was kept constant while the Abs_600_ of the suspension containing the cells treated by PEF decreased. The measurement of the absorbance at 260 and 280 nm revealed no release of UV absorbing material from untreated cells after 8 days of incubation but the amount of UV absorbing material released drastically increased in the samples that contained cells treated by PEF after the same storage period. After 18 days of storage the amount of mannoproteins released from the untreated cell was negligible. Conversely, mannoprotein concentration increased linearly for the samples containing cells of *S. cerevisiae* treated by PEF. After 18 days of incubation the concentration of mannoproteins in the supernatant increased 4.2 times for the samples containing cells treated by PEF at 15 and 25 kV/cm for 45 and 150 μs. Results obtained in this study indicates that PEF could be used in winemaking to accelerate the *sur lie* aging or to obtain mannoproteins from yeast cultures.

## Introduction

Yeast cell wall, which represents up to 20% of yeast cell dry weight, is mainly composed of β-glucans and mannoproteins. These mannoproteins are highly glycosylated (∼90% sugars, mainly mannose) and are located in the outermost layer of the yeast cells acting as structural components (Quiros et al., 2012). Mannoproteins have been associated with positive quality and technological traits of wines. It has been shown that mannoproteins reduce haze formation, prevent the precipitation of tartaric salt, contribute to the mouthfeel, influence the intensity of the aroma of wine and can interact with phenolic compounds, thus improving color stability and reducing the astringency of wine (Pérez-Serradilla and De Castro, 2008). Furthermore, different studies have demonstrated important emulsifying and stabilizing properties of mannoproteins due to the amphipathic structure of their molecule (da Silva Araújo et al., 2014).

Mannoproteins are released from the yeast cell wall during yeast autolysis. Autolysis is a phenomenon that begins with the disorganization of membranous systems (cytoplasmic membrane and other organelle membranes) caused by cell’s death. During autolysis enzymes glucanase and proteinase degrade the cell wall and, as result, the cell wall becomes porous and different compounds such as mannoproteins are released into the surrounding medium (Alexandre and Guilloux-Benatier, 2006).

Releasing of mannoproteins from yeast autholysis occurs during the alcoholic fermentation but mainly during the aging on the lees of certain types of wines such as white, red, or sparkling. Wine lees are a residue that is formed at the bottom of the recipes containing wine after fermentation and that is mainly composed by yeast. The autolysis of yeast in wine is a very slow process lasting from a few months to years. Therefore accelerating this process is highly desirable to reduce the risk of microbial spoilage of wine and decrease production costs (Alexandre and Guilloux-Benatier, 2006; Comuzzo et al., 2015).

Different strategies have been suggested for accelerating yeast autolysis. Enzymes able to hydrolize B-glucans from yeast cell walls and thermolysis are the most widely proposed tools (Martínez-Rodríguez et al., 2001; Comuzzo et al., 2012; Bzducha-Wróbel et al., 2014). Recently the potential of non-thermal processing technologies such as high pressure homogenization (Comuzzo et al., 2015) and ultrasound (Martín et al., 2013) have been also investigated to induce autolysis of wine yeasts.

High pressure homogenization is one of the most commonly employed mechanical methods for large scale disruption of microbial cells. This method results in effective breakage of cells and high recovery of bio-products. However, HPH causes non-selective release of the products and its final products contain large quantity of cell debris which complicates the downstream process of purification (Comuzzo et al., 2015).

Pulsed electric fields (PEF) is a technology that causes loss of the barrier function of the cell membranes by application of intermittent electric fields of high intensity for short periods of time (μs–ms) (Barba et al., 2015; Puértolas and Barba, 2016). The phenomenon, that is called electroporation, is mainly associated to the formation of local defects or pores in the cytoplasmic membrane of the cells increasing its permeability and causing uncontrolled molecular transport across microbial membranes. Recently, it has been reported that PEF provokes not only cytoplasmic membrane permeabilization but also changes in the cell wall structure (Ganeva et al., 2014; Pillet et al., 2016). This technology has been successfully applied to recover different intracellular components such as proteins, nucleic acids, and ionic substances from different yeast species (Ganeva et al., 2003; Liu et al., 2013).

The aim of this study was to evaluate the potential application of PEF to induce accelerate autolysis of a commercial strain of *Saccharomyces cerevisiae* for winemaking use. The effect of PEF treatments of different intensity on cell viability, cytoplasmic membrane permeabilization, and release of mannoproteins and compounds absorbing at 260 and 280 nm has been investigated.

## Materials and Methods

### Strains, Medium, and Propagation Conditions

A strain of *S. cerevisiae* from an industrial preparation for winery applications was used (Levuline Sélection C.I.V.C. France, Bahnhofstrasse, Switzerland). Yeasts were grown in in 1000 mL glass flasks containing 600 mL of Sabouraud-Dextrose broth (Oxoid, Basingstoke, UK) under agitation at 25°C. Yeast’s growth was monitored by measuring the absorbance at 600 nm and the number of cells using a Thoma counting chamber and the plate counting method in Potato-Dextrose-Agar (PDA, Oxoid, Basingstoke, UK). The experiments were performed with cells at stationary growth phase, which was achieved after 48 h of incubation.

### PEF Treatment

The PEF equipment used in this investigation was previously described by Saldaña et al. (2010). Before treatment, fresh biomass of *S. cerevisiae* was centrifuged at 3000 × *g* for 10 min at 25°C and re-suspended in a citrate-phosphate Mcllvaine buffer (pH 7.0; 1 mS/cm) to a final concentration of approximately 10^9^ cells mL^-1^. The *S. cerevisiae* suspension (0.44 mL) was placed in the treatment chamber by means of a 1 mL sterile syringe (TERUMO, Leuven, Belgium). Cells were subjected to 5–80 monopolar square waveform pulses of 3 μs of electric field strengths between 5 and 25 kV/cm at room temperature and applied at a frequency of 0.5 Hz.

### PEF Inactivation

After the PEF treatments, cells were plated in PDA in order to monitor inactivation after different treatment conditions. Serial dilutions were pour plated and the number of viable cells, expressed in colony-forming units (CFU), corresponded to the number of colonies counted after 48 h of incubation at 25°C. Inactivation data was expressed as the ratio between the initial number of survivors (N_o_) and the number of survivors after different treatment times (N_t_).

### Staining Cells with Propidium Iodide

Quantification of the number of *S. cerevisiae* electroporated cells was performed by measuring the entry of the fluorescent dye propidium iodide (PI; Sigma-Aldrich, Barcelona, Spain). PI is a small (660 Da) hydrophilic molecule that is unable to cross through intact cytoplasmatic membranes. 50 μL of PI (0.1 mg mL^-1^) were added to 450 μL of *S. cerevisiae* suspension, resulting in a final concentration of 0.015 mM. After the PEF treatments, suspensions were incubated for 10 min. Previous experiments showed that longer incubation times did not influence the fluorescence measurements. After incubation the cell suspension was centrifuged and washed two times until no extracellular PI remained in the buffer. PI trapped inside the cells was quantified by spectrofluorophotometry. Results were expressed as the percentage of permeabilized cells based on the fluorescence value obtained for cells permeabilized by the most intense PEF treatment (240 μs at 25 kV cm^-1^) used in this investigation. Under these conditions, the permeabilization of individual cells was also checked using an epi-fluorescence microscope (Nikon, Mod. L-Kc, Nippon Kogaku KK, Japan). Fluorescence was measured with a spectrofluorophotometer (mod. Genios, Tecan, Austria) using 535-nm excitation filter (523–547 nm) and a 625-nm emission filter (608–642 nm). Two alternative staining protocols were followed under the same experimental conditions to detect reversible and irreversible electroporation.

#### Staining Cells before PEF Treatments

When PI was added before PEF treatments stain cells corresponded to the sum of both the irreversibly and reversibly permeabilized cells.

#### Staining Cells after PEF Treatments

The degree of permeabilization when cells were stained after the PEF treatment corresponded to irreversibly permeabilized cells. Reversible permeabilization was calculated by comparing the fluorescent measurements obtained following the two staining protocols.

### Storage of Cellular Suspensions and Determination of Yeast Viability

Control and PEF treated cells were re-suspended in buffer of pH 7.0 and stored at 25°C. Samples were collected at different time points along the period of storage which lasted 25 days.

The viability of cells during the storage was determined by pour plating of serial dilutions and counting the colony-formed after 48 h of incubation at 25°C.

### Monitoring Cell Lysis Caused by PEF

In order to monitor the release of components during storage of cellular suspensions, different measurements were performed in untreated and PEF treated samples.

Turbidity of the suspension during the storage was measured by the absorbance at 600-nm (Abs_600_) to monitor leakage of cellular content. Absorbance at 260-nm (Abs_260_) and 280-nm (Abs_280_) of the supernatant was measured in order to monitor the presence of intracellular material outside the cell (Aronsson et al., 2005).

The concentration of mannoproteins in the extracellular medium was determined after hydrolyzing the supernatant with sulfuric acid (final concentration 1.5 M) at 100°C for 90 min. Cooled samples were neutralized with NaOH 3 M. Quantitative analysis of mannose was conducted by an enzymatic method (D-Mannose, D-Fructose, and D-Glucose assay procedure, Megazyme International, Wicklow, Ireland) (Dupin et al., 2000).

### Statistical Data Treatment

The results represent the mean ± standard error of the mean of three replicates. One-way ANOVA test was conducted to assess significant differences between treatments. The differences were considered significant at *p* < 0.05.

## Results and Discussion

### PEF Inactivation of *S. cerevisiae* as Function of the Electric Field Strength and Treatment Time

**FIGURE 1:**
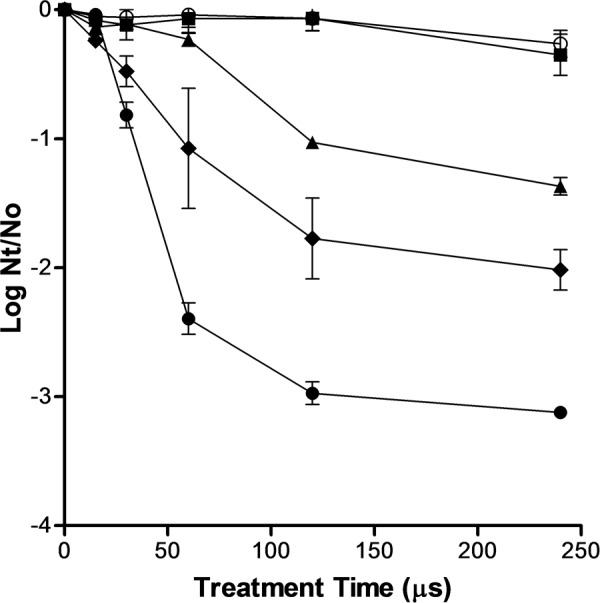
**Inactivation of *Saccharomyces cerevisiae* by pulsed electric fields (PEF) treatments of different electric field strengths.** 5 kV/cm (○), 10 kV/cm (▪), 15 kV/cm (▲), 20 kV/cm (◆), 25 kV/cm (●). Inactivation data was expressed as the ratio between the initial number of survivors (N_o_) and the number of survivors after different treatment times (N_t_).

The inactivation curves of *S. cerevisiae* after exposure to PEF treatments of different electric field strengths and duration is shown in **Figure 1**. It can be observed that treatments below 10 kV/cm were ineffective to inactivate *S. cerevisiae*. These results confirms data obtained by other authors showing that electric field higher than 10 kV/cm were required to inactivate different types of yeast when pulses of a duration of microseconds were applied (Cserhalmi et al., 2002; Aronsson et al., 2005). Currently, it is accepted that the main mechanism involved in microbial inactivation by PEF is electroporation that is is a consequence of an increment in the transmembrane voltage (Heinz et al., 2001). The external electric field strength required to reach the transmembrane voltage threshold to induce electroporation is correlated with the cell size (Heinz et al., 2001). This dependence explains why the critical electric field required to electroporate yeast is lower than that required to electroporate bacteria -which size is lower- and higher than that required to electroporate eukaryotic cells of plants or animal tissues -which size is higher-.

As it has been reported by other authors, above the critical electric field strength *S. cerevisiae* inactivation increased with more intense electric field strength and longer treatment durations (Saldaña et al., 2014). However, the inactivation kinetics of *S. cerevisiae* was non-linear. Thus, at any electric field strength assayed, the inactivation was faster in the first moments of the treatment and then the number of survivors decreased more slowly as the treatment time increased. A treatment of 120 μs (40 pulses of 3 μs) inactivated around 1.0, 1.7, and 2.7 log cycles the population of *S. cerevisiae* when applied at 15, 20, and 25 kV/cm, respectively (**Figure 1**). Nevertheless, a further increment of treatment duration from 120 to 240 μs scarcely increased the lethality of PEF.

### PI Entry into *S. cerevisiae* Cells as Function of the Electric Field Strength and Treatment Time

**FIGURE 2:**
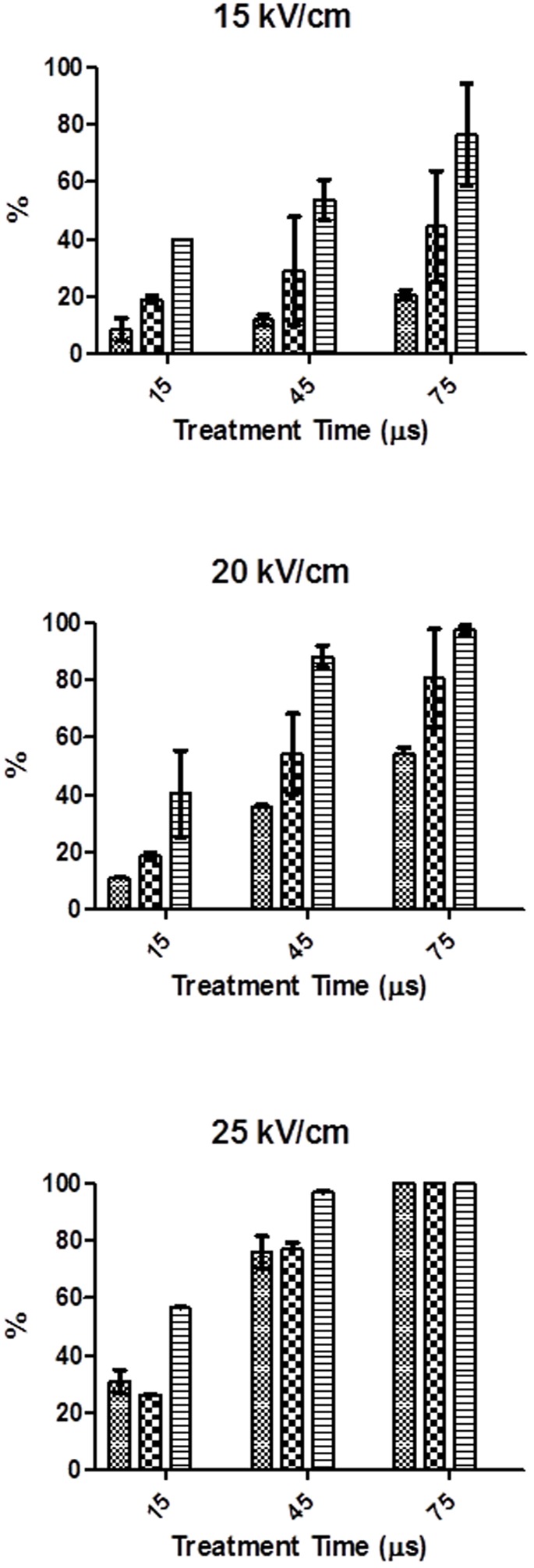
**Percentage of *S. cerevisiae* cells inactivated and stained when propidium iodine (PI) was add before or after PEF treatments of different electric field strength and treatment time.** 512 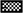 % of stained cells when PI added after PEF treatment, 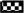 % of stained cells when PI added before PEF treatment, 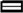 % of inactivated cells.

**Figure 2** shows the percentage of cells permeabilized to PI after PEF treatments of different electric field strength and duration when PI was added before or after the treatment. For comparison purposes, the percentage of *S. cerevisiae* cells inactived by the same PEF treatments are also shown in **Figure 2**. As it can be observed in the figure, the entry of PI increased with the treatment time and intensity of the electric field strength, regardless of the staining protocol. In order to detect significant permeabilization to PI an electric field strength equal or higher than 15 kV/cm was required. At electric field strengths of 15 and 20 kV/cm, the difference between the PI entry observed by the different staining protocols under the same PEF treatment conditions reveals the existence of reversible electroporation. It means that in a proportion of cells, the permeabilization caused by PEF disappeared after the treatment. However, at the highest electric field strength assayed (25 kV/cm) all the population was irreversibly electroporated. This dependence between the intensity of the electric field strength and the proportion of cells reversibly electroporated has been previously observed by other authors in other microorganisms such as bacteria (García et al., 2007; Cebrián et al., 2015), microalgae (Luengo et al., 2014) and other species of yeasts (Aronsson et al., 2005).

According to **Figure 2**, the number of irreversibly permeabilized yeast cells was, in general, lower than the number of dead cells but the difference decreased for the PEF treatments applied at higher intensity. These results indicate that a percentage of yeasts cell that are inactivated during the treatment was able to recover the integrity of the membrane-becoming the cytoplasmatic membrane not permeable to PI when the dye was added after the treatment- or that death of these cells could be caused by secondary damages to other structures or functions (Aronsson et al., 2005; García et al., 2007). Other authors have also observed the presence of dead microbial cells of bacteria and microalgal with non-permeabilized cytoplasmatic membranes after the application of PEF treatments at moderate intensity (García et al., 2007; Luengo et al., 2014; Cebrián et al., 2015). As it was also reported by these authors our results confirm that after applying intense PEF treatments (25 kV/cm for 75 μs) no difference between the percentage of *S. cerevisiae* cells inactivated and irreversively electroporated was observed.

### Decrease of the Optical Density and Leakage of Intracellular Material after Application of PEF Treatments

Decrease in the absorbance at 600 nm and presence of UV absorbing material in the suspension medium were used as indicators of the degree of cell lysis caused by PEF. Thus, when the permeability of the cytoplasmatic membrane of the microorganism is altered water diffuses from the external medium to the cytoplasm causing a decrease in the optical density of the cell suspension. On the other hand, the presence of intracellular material outside the cell can be detected by measuring the absorbance of the suspending medium at 260 and 280 nm, which corresponds with the absorbance maxima of nucleic acids and proteins, respectively.

**FIGURE 3:**
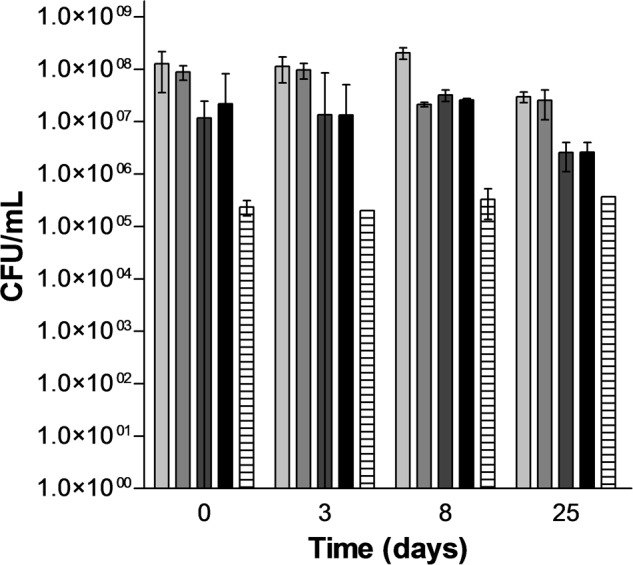
**Evolution of the population of untreated and PEF treated cells of *S. cerevisiae* along the incubation time.** 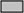 Untreated, 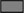 15 kV/cm 45 μs, 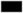 15 kV/cm 150 μs, 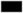 25 kV/cm 45 μs, 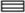 25 kV/cm 150 μs.

In order to evaluate the potential of PEF for inducing lysis of *S. cerevisiae*, four treatments of different intensity were selected: a treatment that inactivated around 50% of S. *cerevisiae* (15 kV/cm, 45 μs), two treatments that inactivated around a 90% the population of *S. cerevisiae* at low (15 kV/cm, 150 μs) and high (25 kV/cm, 45 μs) electric fields and a treatment that inactivated around 99.9% the population of *S. cerevisiae* (25 kV/cm, 150 μs). **Figure 3** illustrates the inactivation obtained just after the PEF treatment (time 0) and the viability of the PEF treated *S. cerevisiae* cells along the incubation time. Statistically significant differences (*p* < 0.05) in the survivor number were not observed in the control cells and in cells treated with the most intense PEF treatments (15 kV/cm, 150 μs and 25 kV/cm for 45 and 150 μs) after 25 days of incubation. Conversely, the population of the *S. cerevisiae* cell treated at 15 kV/cm for 45 μs decreased progressively from day 3 to day 8 of incubation. Thus, after 8 days of incubation the number of viable cells in this suspension was similar to the number of viable cell in the suspensions containing cells treated at 15 kV/cm for 45 μs and 25 kV/cm for 150 μs. These results indicate that when PEF treatments are applied at low intensities a proportion of the population is injured rather than inactivated. Since incubation of the microorganisms in a buffer of pH 7.0 is not an optimal recovery condition, subletally injured cells of *S. cerevisiae* would not be able to repair this damage and they would dead during incubation. Inactivation by a subsequent incubation under non-favorable conditions of yeast and bacteria treated by PEF treatments of moderate intensity that did not cause a significant inactivation has been previously observed by other authors (Somolinos et al., 2007, 2008).

**FIGURE 4:**
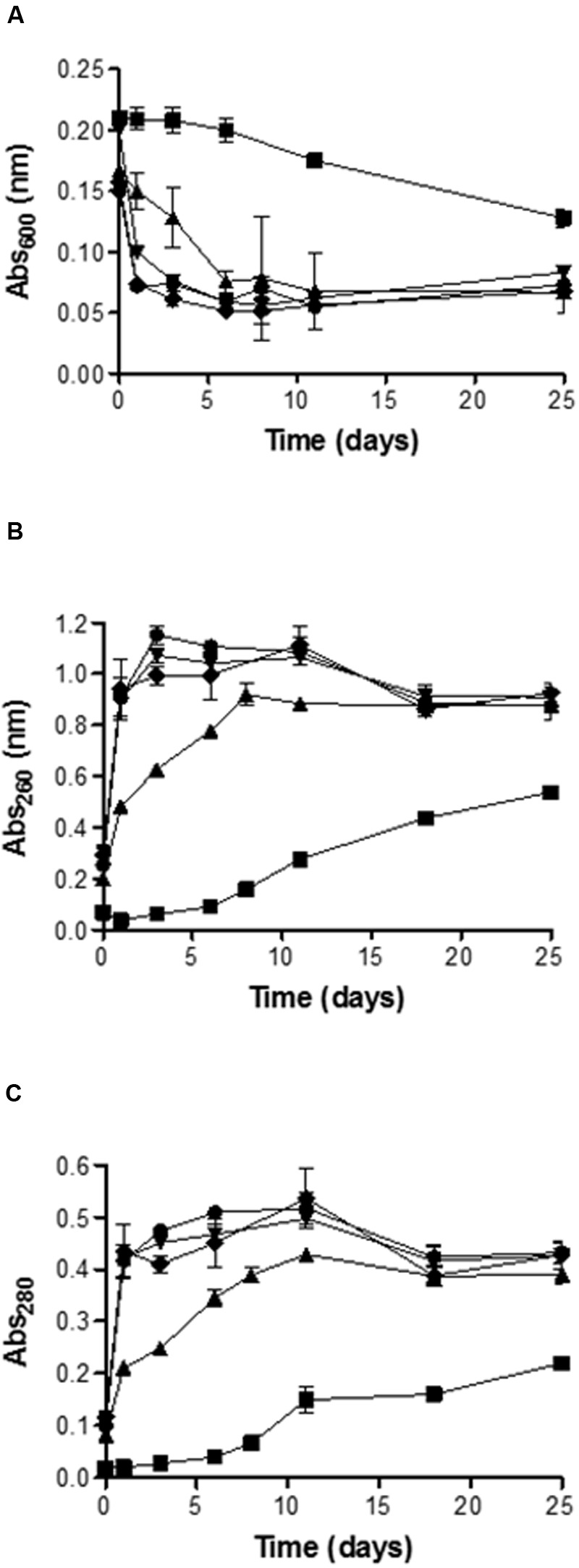
**Evolution along the time of the absorbance at 600-nm **(A)**, 260-nm **(B)** and 280-nm **(C)** of the medium containing untreated and PEF treated cells of *S. cerevisiae.*** Untreated (▪), 15 kV/cm 45 μs (▲), 15 kV/cm 150 μs (▼), 25 kV/cm 45 μs (◆), 25 kV/cm 150 μs (●).

**Figures 4A–C** shows the evolution along the time of the absorbance at 600 nm of the yeast suspension and of UV absorbing material of the suspension medium at 260 and 280 nm, respectively, after the application of the PEF treatments.

**Figure 4A** shows that the decrease of abs_600_ of the yeast suspension was a function of the intensity of the PEF treatment applied. After 24 h of incubation the absorbance of the suspension containing untreated yeast was maintained constant: By contrast, the absorbance of the suspensions containing PEF treated yeast decreased around 25% for the cells exposed to treatment of 15 kV/cm for 45 μs and 62.5% for the cells treated at 15 kV/cm for 150 μs. Differences statistically not significant (*p* < 0.05) were observed in the abs_600_ decrease between this last treatment and both treatments applied 25 kV/cm. On the other hand, it should be noted that while further incubation of the suspensions containing cells treated at 15 kV/cm for 150 μs or at 25 kV/cm resulted in almost no changes in their abs_600_, the abs_600_ of the suspension containing cells treated at 15 kV/cm for 45 μs progressively decreased until reaching the same value as the OD of the rest of suspensions after 6 days of incubation. After 25 days of incubation the decrease in abs_600_ of the suspension containing control cells was still 50% lower than the abs_600_ of the suspension containing cells treated by PEF.

Measurement of UV absorbing substances at 260 and 280 nm was used as an index of the amount of intracellular components (mainly nucleic acids and proteins) leaking from cells after exposure to PEF. In the first moments after the treatment, the leakage of UV absorbing components as measured at both wavelengths was higher after the treatments carried out at higher electric field strength. The less severe treatment (15 kV/cm, 45 ms) resulted in an increase in 0.5 and 0.2 absorption units at 260 and 280 nm, respectively, after 24 h. By contrast, the rest of the treatments yielded increases of 0.95 and 0.43 units at 260 and 280 nm, respectively, and the absorbance values reached were maintained almost constant during all the incubation time.

Regarding the evolution of the absorption values at 260 and 280 nm of the medium containing the yeast cells treated at the lowest PEF treatment intensity, the values progressively increased until days 8–10 of incubation. The time required to reach the maximum absorbance values also corresponded with the time of incubation required for the death of cells treated at 15 kV/cm for 45 μs. Therefore, inactivation of the sublethal injured cells was accompanied by the release of nucleic acids and proteins to the extracellular environment. These observations suggest that PEF treatment applied in this investigation caused the formation of pores large enough to permit the leakage of molecules such as proteins that are much bigger that PI. On the other hand, results obtained indicate that the amount of molecules leaked was correlated with the proportion of dead cells in the suspension. However, no significant differences were observed in abs_600_ decrease or leakage of nucleic acids and proteins when the proportion of dead cells in the suspension was higher than the 90%.

### Release of Mannoproteins to the Extracellular Environment as Function of the Intensity of the Electric Field Strength Treatment

**FIGURE 5:**
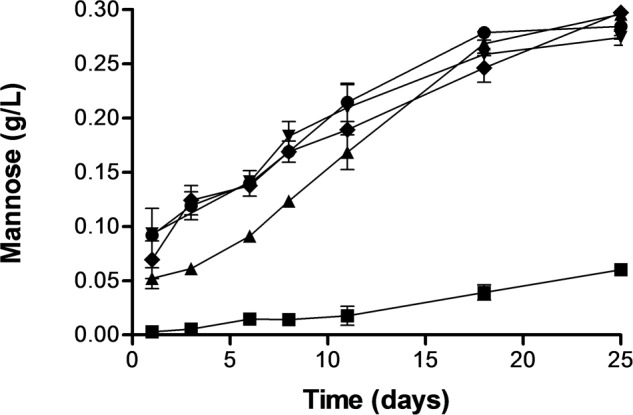
**Release of mannose from *S. cerevisiae* cells untreated and treated by PEF treatments of different intensity.** Untreated (▪), 15 kV/cm 45 μs (▲), 15 kV/cm 150 μs (▼), 25 kV/cm 45 μs (◆), 25 kV/cm 150 μs (●).

Release of mannoproteins to the extracellular media from suspensions containing untreated cells and PEF treated cells at the same treatment intensities described above was monitored by determining the mannose concentration of the supernatant after hydrolyzing the polymeric forms into monomeric sugar by addition of sulfuric acid. **Figure 5** shows that the concentration of polymeric mannose into the extracellular environment increased drastically along the time for the samples containing cells treated by PEF but mannose was hardly detected in the suspension containing untreated cells. After 25 days of incubation the concentration of mannose in the samples containing PEF treated cells of *S. cerevisiae* was 10 times higher than in the control. On the other hand, release of mannose in the sample containing cells of *S. cerevisiae* treated at 15 kV/cm for 45 μs was lower than in the rest of the samples containing cells treated by more intense PEF treatments. However, after 18 days of incubation no statistically significant differences (*p* < 0.05) in the polymeric mannose concentration were observed in all samples containing cells treated by PEF. Polymeric mannose release was a process slower than the decrease of the OD or the release of UV absorbing substances. In the samples treated by PEF the concentration of polymeric mannose in the extracellular environment clearly increased until the 18 days of incubation and them concentration remained almost constant.

The process of natural yeast autolysis begins as consequence of the disorganization of membranous systems of the cell, such as the cytoplasmic membrane and other organelles when that occurs with the death of the cell. This permits the endogenous enzymes to come in contact with cellular constituents which are degraded and render soluble. The enzymes glucanase and protease play a significant role in the degradation of cell wall constituents of the yeast and as consequence the cell wall becomes porous and mannoproteins, among other cell wall constituents, are released into the surrounding medium (Alexandre and Guilloux-Benatier, 2006). Results obtained in this investigation confirm that natural autolysis is a slow process (Pérez-Serradilla and De Castro, 2008). Conversely, the electroporation of the yeast by PEF induced autolysis of the cells and a significant amount of mannoproteins were detected in the extracellular medium after only 24 h of incubation. Several mechanism related to electroporation could be involved in the induced autolysis by PEF. On the one hand, electroporation causes a water inlet in the cytoplasm, what has been demonstrated by the increment of the absorbance at 600 nm of the suspension. The decrease of the osmotic pressure in the cytoplasm as consequence of the water inlet could cause the plasmolysis of the organelles and the release of the enzymes. On the other hand, the electroporation of the cytoplasmic membrane by PEF could facilitate the contact of these enzymes with the outermost layer of the yeast cell wall were the mannoproteins are located.

According to observation in this investigation, PEF could be used in winemaking to accelerate the *sur lie* aging reducing the risk of microbial spoilage by yeast such as *Brettanomyces* and biogenic amine contamination or to obtain mannoproteins from yeast cultures to be used in winemaking. Furthermore, mannoproteins obtained by PEF induced yeast autholysis could be used for other applications in the food industry because these molecules have interesting emulsifying and stabilizing properties due to the amphipathic structure of the mannoprotein molecule (da Silva Araújo et al., 2014).

## Conclusion

Results obtained in this study show the potential of PEF to induce autolysis in *S. cerevisiae* cells and to accelerate the release of mannoproteins to the extracellular medium. The major advantage of PEF, as compared to other process such as thermolysis, is that the lytic process occurs without thermal damage, thus avoiding the formation of odorant compounds reported by other authors when high temperatures are applied during the processing of yeast-derivated products (Münch and Schieberle, 1998; Pozo-Bayón et al., 2009).

## Author Contributions

JM: substantial contributions to the acquisition and analysis, data for the work; drafting the work; final approval of the version to be published; agreement to be accountable for all aspects of the work in ensuring that questions related to the accuracy or integrity of any part of the work are appropriately investigated and resolved. GC: substantial contributions to the acquisition, analysis, data for the work; drafting the work or revising it critically for important intellectual content; final approval of the version to be published; agreement to be accountable for all aspects of the work in ensuring that questions related to the accuracy or integrity of any part of the work are appropriately investigated and resolved. IA: substantial contributions to the conception or design of the work; drafting the work or revising it critically for important intellectual content; final approval of the version to be published; agreement to be accountable for all aspects of the work in ensuring that questions related to the accuracy or integrity of any part of the work are appropriately investigated and resolved. JR: substantial contributions to the conception or design of the work; analysis, and interpretation; drafting the work or revising it critically for important intellectual content; final approval of the version to be published; agreement to be accountable for all aspects of the work in ensuring that questions related to the accuracy or integrity of any part of the work are appropriately investigated and resolved.

## Conflict of Interest Statement

The authors declare that the research was conducted in the absence of any commercial or financial relationships that could be construed as a potential conflict of interest.
